# Toward Integrative Management Advice of Water Quality, Oil Spills, and Fishery in the Gulf of Finland: A Bayesian Approach

**DOI:** 10.1007/s13280-013-0482-7

**Published:** 2014-01-12

**Authors:** Mika Rahikainen, Inari Helle, Päivi Haapasaari, Soile Oinonen, Sakari Kuikka, Jarno Vanhatalo, Samu Mäntyniemi, Kirsi-Maaria Hoviniemi

**Affiliations:** 1 Fisheries and Environmental Management Group (FEM), Department of Environmental Sciences, Helsinki University Centre for Environment (HENVI) and Finnish Environment Institute (SYKE), Viikinkaari 2a, P.O. Box 65, 00014 Helsinki, Finland; 2 Fisheries and Environmental Management Group (FEM), Department of Environmental Sciences, University of Helsinki, Viikinkaari 2a, P.O. Box 65, 00014 Helsinki, Finland; 3 Finnish Environment Institute (SYKE), Finnish Game and Fisheries Research Institute, MTT Agrifood Research Finland, Latokartanonkaari 9, 00790 Helsinki, Finland

**Keywords:** Baltic Sea, Bayesian networks, Probabilistic model, Uncertainty, Environmental management, Integrated risk analysis

## Abstract

Understanding and managing ecosystems affected by several anthropogenic stressors require methods that enable analyzing the joint effects of different factors in one framework. Further, as scientific knowledge about natural systems is loaded with uncertainty, it is essential that analyses are based on a probabilistic approach. We describe in this article about building a Bayesian decision model, which includes three stressors present in the Gulf of Finland. The outcome of the integrative model is a set of probability distributions for future nutrient concentrations, herring stock biomass, and achieving the water quality targets set by HELCOM Baltic Sea Action Plan. These distributions can then be used to derive the probability of reaching the management targets for each alternative combination of management actions.

## Introduction

In 2007, the Helsinki Commission (HELCOM) launched the Baltic Sea Action Plan (BSAP) which, by focusing on eutrophication, hazardous substances, biodiversity, and maritime activities, aims at restoring the good ecological status of the Baltic marine environment by 2021. The aims of the BSAP are supported by the EU Strategy for the Baltic Sea region, which addresses the environmental challenges of the sea through intensifying cooperation in the region and implementing the Integrated Maritime Policy in the Baltic (CEC 2009). The focus of the Integrated Maritime Policy is in the coordination of interrelated issues: for instance, the relationship between improvements to the sea quality and increased employment in terms of better marine business potential using the EU green growth initiative as catalyst.

Achieving good environmental status calls for the incorporation of the latest scientific knowledge and innovative management approaches into strategic policy implementation. This requires an understanding on the components, dynamics, and interactions of the complex ecosystem, and how it reacts to anthropogenic pressures. In addition to understanding the past and present state of the ecosystem, information is needed for managing the future state of the system. In large scale environmental problems, the management targets are typically set for 10–20 years. Consequently, predictions are of paramount importance, but they will be uncertain because of the stochasticity of natural systems and limitation of the current knowledge. As the Baltic Sea ecosystem is altered by several stressors at the same time, integrated analyses are needed.

The cause–effect relationships between the anthropogenic stressors and harmful environmental effects should be analyzed in a manner enabling decision makers to consider the risk level of the decisions made. Technically risk is defined as the product of a probability of something environmentally harmful happening and the consequence of such event. Thus, if the research community provides a single estimate, decisions will essentially be based on overconfident information. For instance, the prediction may indicate that given an action, policy target will not be met since the predicted value is below the target value. In contrast, if decision makers are provided results in the form of a probability distribution, they get more honest information (Mäntyniemi et al. 2009). If a probability distribution is provided instead of a single estimate, then this can, for example, indicate 60 % chance for meeting the target. Probabilities are highly useful when alternative actions are ranked in a decision analytic framework.

There are two approaches to produce statistical inference: the classical frequentist and the Bayesian. The key difference between them in the context of environmental problems is: (1) because the frequentist approach deals only with the uncertainty about potentially observable data, it does not allow assessing uncertainty about states of nature, but (2) Bayesian approach explicitly includes knowledge in the form of a probability statement about states of nature. We advocate the Bayesian approach since it enables the use of existing information that can be updated with new information. Bayesian Belief Networks (BBNs) also are flexible in combining different risk perspectives. The technical risk definition can be effortlessly combined with the economic perspective of agents aiming at utility maximization and/or sociocultural risk definition according to which social groups assign meaning to an environmental harm (Renn 2008).

Bayesian belief networks are graphic models that enable linking several risk factors and their management options in one model, and the examination of their impact on variables of management interest (Jensen 2001). In studies aiming at solving the Baltic Sea environmental problems, BBNs have been applied to oil spill and other environmental risk assessments (Kuikka et al. 1999; Helle et al. 2011; Lecklin et al. 2011), to fish stock assessments (Mäntyniemi et al. 2013a), and for decision analyses (Varis and Kuikka 1999; Levontin et al. 2011; Lehikoinen et al. 2013), also involving human perspectives (Haapasaari et al. 2007; Haapasaari and Karjalainen 2010). The probabilistic knowledge used in BBN models has been based on the estimation of probabilities with various statistical methods and expert knowledge (Uusitalo et al. 2005). Also, participatory modeling has been facilitated by BBNs (Haapasaari et al. 2012, 2013; Mäntyniemi et al. 2013b).

**Fig. 1:**
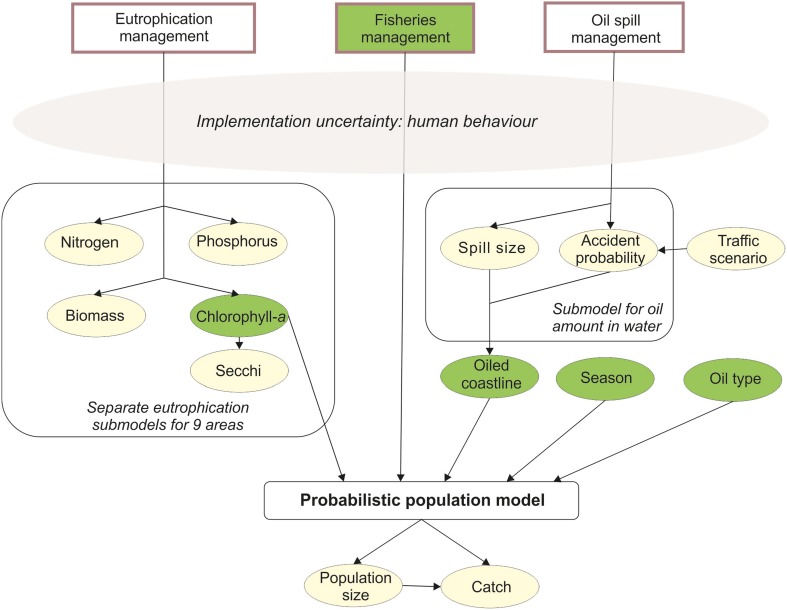
The stylized structure of the Bayesian decision model for the GoF management advice. *Rectangles* and *ellipses* represent decision and random variables, respectively. The *green variables* are inputs from other models to the population dynamic model for herring. The influence of the actions depends on the environmental stochasticity, uncertainty in knowledge, and on the strength of the dependencies between actions and response. The full submodel related to oil spills has 16 variables, and submodels related to eutrophication 3–9 variables, depending on the area of interest in the GoF

The IBAM project (Integrated Bayesian risk analysis of ecosystem management in the Gulf of Finland) studied several anthropogenic pressures that affect the ecosystem of the Baltic Sea, using an integrative Bayesian decision model. The project focused especially on the Gulf of Finland. In this article, we describe two subcomponents of the grand model (Fig. 1) in more detail,: the water quality modeling, and herring analysis. The former is an example of modifying deterministic model to provide probabilistic output. The latter illustrates how multiple stressors are combined while taking uncertainty explicitly into account.

First, we present the BBN approach. Second, we give a short description of the integrative decision model, after which we describe in more detail the water quality modeling and herring analysis, concentrating on the impact of eutrophication and oil spills on the herring stock dynamics. Then, we provide some example results and discuss the advantages and challenges related to the work. Finally, we conclude by discussing the relevancy of Bayesian modeling approach to research and management.

## Bayesian Networks in Integrated Risk Analysis

The Bayesian theory allows learning as a process in which humans constantly update their understanding of the world. In practice, problems are often structured into graphic cause–effect relationships which permit examining how an information change in one variable affects that of the other ones. The Bayes’ theorem is used to update the preunderstanding (prior knowledge) of a problem by new information, to get a novel understanding (posterior knowledge) of the issue (Pearl 1988; Spiegelhalter et al. 1993; Dennis 1996). The strength of the links between variables is expressed by conditional probability distributions. The more uncertain the relationship between the variables is the wider is the probability distribution. As knowledge accumulates so that our uncertainty about the phenomenon and parameters decreases, also the probability distribution narrows. This way the Bayesian approach describes uncertainty in an explicit manner.

The Bayesian approach is based on subjective knowledge. Thus, a real-world problem structured into a Bayesian model is based on the researcher’s interpretation of the existing knowledge related to the problem. The knowledge can originate from new experimental data, the literature, preexisting models, or statistics. It can also be elicited from scientific or other competent experts. In most cases, “expert knowledge” refers to knowledge elicited from a scientific expert, in relation to a model structure or probability distribution, or both. In the subjective terms, the probability is expressed as a *degree of belief* which means a private assessment of how likely an event is, based on the available evidence (Ramsey 1926; Spiegelhalter et al. 1993; Gelman et al. 1995; Nau 2001). While formulating subjective probabilities is one of the practical challenges of the Bayesian approach, they make a consistent combination of different types of information possible. The subjectivist Bayesian approach differs fundamentally from the frequentist inference that builds on the ideal of objectivity, unbiased analyses, experimental evidence, and infinite sampling (Malakoff 1999). A Bayesian Belief Net consists of uncertain variables. By adding variables that can be controlled (managerial decisions) and variables that measure utility or loss (i.e., preference) related to uncertain variables, the impact and utility of the management measures can be evaluated.

## Building a Bayesian Decision Model for the Gulf of Finland

The integrative decision analysis model encompasses three risk factors present in the Gulf of Finland (hereafter GoF): eutrophication, unsustainable fishing, and oil spills (Fig. 1). The eutrophication part of the integrative BBN model can be used to assess the probability of reaching the water quality targets set by the EU’s Water Framework Directive (WFD) for different types of the coastal waters (Aroviita et al. 2012) or by the Helsinki Commission Baltic Sea Action Plan (HELCOM BSAP) for the open sea (HELCOM 2012), and it also includes a variable describing the overall chlorophyll status, linked to the herring stock dynamics. The rest of the model includes the dynamics related to oil spills and harvesting. The oil spill component of the model is partially based on a previous project which studied the risks related to maritime traffic in the GoF (Klemola et al. 2009). The final output nodes are variables describing the abundance of the herring and catch by the commercial fishery. Various techniques and models were used to produce conditional probability tables for the random variables included in the model. These include outputs from a three-dimensional (3D) ecosystem model, probabilistic population dynamic models, and expert knowledge.

### Predicting Nutrient Concentrations in the GoF

The main aim of water quality-modeling efforts was to offer a way to assess how successful different loading reduction scenarios could be in meeting the water quality targets of the WFD and HELCOM BSAP. The integrative model includes five water quality variables: nitrogen, phosphorus, phytoplankton biomass, chlorophyll-*a*, and Secchi depth, which can be studied in nine areas within the GoF. Finnish and Estonian coastal areas were defined according to the national implementation of the WFD, and the division of the open sea areas was based on a previous similarity analysis.

Six nutrient loading scenarios were included in the analysis: Business-as-usual (BAU, i.e., no additional measures taken compared with the situation in early 2000s) for Finland, Estonia, and Russia, 100 and 50 % implementation of the Baltic Sea Action Plan for all three countries, and three alternative reduction scenarios only for Finland.

The influence of external loads to the water quality in GoF is currently described by the 3D EIA-SYKE ecosystem model (Kiirikki et al. 2001, 2006). This model is deterministic providing point estimates, while Bayesian decision analysis uses probabilistic information. In order to use deterministic results in a probabilistic framework, a Gaussian Process approach was used to extend the deterministic ecosystem model into a probabilistic form (Vanhatalo et al. 2013).

### Gulf of Finland Herring Stock Dynamics and Fishery

Herring is one of the key species in the Baltic ecosystem due to its high abundance and its role in the pelagic food web (Sparholt 1994; Flinkman et al. 1998; Kornilovs et al. 2001; Mäntyniemi et al. 2012; Suuronen and Lehtonen 2012). Herring stocks also provide the most valuable fishery in Finland (Finnish Game and Fisheries Research Institute 2004). Thus, there are abundant data and knowledge about the Baltic herring, which simplify estimating the current state of the stock and simulating its future development.

In IBAM, a probabilistic population dynamics model was developed for the GoF herring stock (Rahikainen et al., unpublished). The model included the relevant population dynamics variables and their dependencies in a probabilistic form. The population model incorporated different harvest mortalities, the effects of oil on juveniles and adults, the influence of eutrophication on the recruitment of the herring, i.e., the risk factors included in the integrative decision model.

More specifically, information about chl-*a* concentration, sea surface temperature, salinity, and abundance of sprat (*Sprattus sprattus*) and cod (*Gadus morhua*) were used as explanatory variables to account for their impact on herring recruitment, growth rate, and natural mortality rate. In this model, chl-*a* concentration stands for effects of eutrophication. A Gaussian Process approach was used to produce probabilistic estimates about chl-*a*, sea surface temperature, and salinity. Moreover, the possible additional mortality caused by the tanker Antonio Gramsci oil spill in the GoF in 1987 was included in the model by means of previously published probabilistic knowledge of the oil-induced mortality on pelagic fishes (Lecklin et al. 2011) as prior information.

After finishing the population model, it was used to simulate the future states of the herring population and catch by applying different eutrophication levels (resulting from nutrient-loading scenarios), fishing mortalities, and the effect of uncertain oil spills, after which the results were fed into the integrative decision model.

## Example Results and Pros and Cons of the Bayesian Decision Modeling Approach

### Is Water Quality Going to be Better and Herring Stock Larger?

The outcome of the integrative Bayesian decision model is a set of explicit distributions for the model variables, such as nutrient concentration and the herring stock biomass in the GoF. These distributions also indicate probabilities of instances, for example, meeting the water quality targets under the alternative nutrient load reduction scenarios. In the following, we present and discuss some example results that can be produced with the model.

**Fig. 2:**
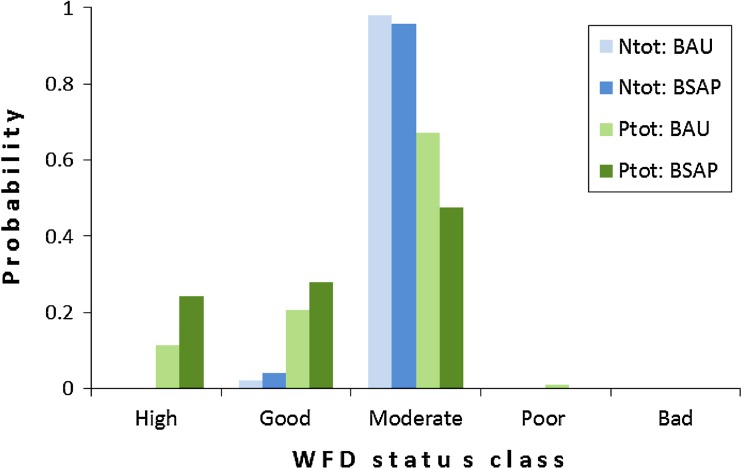
An example of the results of the probabilistic water quality modeling. The *columns* illustrate the probability that the variable is in a certain class defined according to the WFD in the Estonian eastern coastal waters. The class boundaries are from Anonymous (2009). *N*_tot_ and *P*_tot_: Total nitrogen and total phosphorus, respectively; *BAU* and *BSAP* business-as-usual and Baltic Sea Action Plan nutrient loading scenarios, respectively

An example of water quality-modeling results is presented in Fig. 2, which indicates the probability of nutrient concentrations being in a certain quality class in the eastern Estonian coastal waters. The figure underlines several issues. First, BSAP has a positive effect on the water quality, as it shifts the probability distributions toward better classes. Second, the probability to reach “Good” or “High” status varies among variables, and the probability of reaching the target classes is higher (i.e., there is more probability mass in the higher classes) for phosphorus than nitrogen. Third, uncertainty varies between variables and is larger for *P*_tot_ (i.e., the distribution is wider) compared with *N*_tot_.

Regarding the GoF herring, nutrient reduction policies have a minor effect on population abundance at all alternative oil spill and fishery management scenarios. Even the most effective nutrient reduction scenario shifts the probability mass of the herring abundance just slightly toward upper classes. This is an outcome of the fact that the predicted change in the chl-*a* level, impacting herring recruitment, will be minor. Maritime safety tools are not useful either, to manage herring stock abundance (Helle et al., unpublished). It is vital to notice that the results do not indicate that the actual water quality or an oil spill would not affect herring stock size. Instead, major changes in water quality or oil spill frequency cannot to be anticipated. Additional causes to the faint response in herring abundance are stochastic fluctuations in stock dynamics and large posterior uncertainty about the variables.

In the model, fisheries management is expressed via different fishing mortalities that the commercial fleet is allowed to exercise on the herring stock. Controlling fishing mortality notably influences herring abundance, in contrast to nutrient load and oil spill management. The closure of fishing will result in larger population size, whereas doubling the fishing mortality will end in a much lower population level (Helle et al., unpublished). It thus appears that the society’s ability to manage the herring population in the GoF is only effective in the conventional fishery management context, where the bottomline is control of mortality and survival of fishes.

### Why Probabilistic Modeling?

Modeling was carried out by applying methods that allow for uncertainty explicitly. Such an approach has many advantages but also challenges that need to be considered carefully. Vanhatalo et al. (2013) compared the point estimate results of the deterministic EIA-SYKE model with the probabilistic results that were produced by applying the statistical correction procedure to the deterministic model. They conclude that major reductions in nutrient loads are required to achieve the goals of the EU’s WFD or the HELCOM BSAP in the GoF. Noteworthy, they also demonstrated how deterministic models can produce inadequate results for decision-making. As deterministic models offer only a single estimate, they diminish the information decision makers can use, and also eliminate decision-makers’ opportunities to weight their perspective against different levels of uncertainty. This addresses the problematic dichotomy assigned with deterministic models—in plain language, the “answer” for question like “Will the water quality targets be met,” can only be “yes” or “no.” Vanhatalo et al. (2013) reported that when the deterministic model predicts that, for instance, the water quality targets are not met (i.e., the predicted value is below the target value), the probability of success can in reality vary from 0 to as high as 0.51.

However, probabilistic models are capable of advising the decision makers with the probability of achieving the target (Fig. 2; Table 1). For instance, in the eastern Finnish outer archipelago, it is highly likely that targets regarding dissolved inorganic nitrogen (DIN), chl-*a* concentration, and phytoplankton biomass will not be met, while the Secchi depth target will be achieved (Table 1), whatever environmental policy is chosen. Importantly, the probability of achieving the dissolved inorganic phosphorus (DIP) target is 6–21 %, depending on the nutrient reduction policy. A deterministic approach is unable to calculate these probabilities and to indicate that there still is a small chance of reaching the target. Moreover, even though FIN3 scenario reduces Finland’s phosphorus load by 28 % compared with BAU scenario, the probability to achieve WFD target is practically the same under both scenarios. This result is reasonable since in early 2000s, Finland was responsible only for 8 % of the estimated bioavailable phosphorus loads to the GoF, and thus, even the substantial reduction by Finland alone would have negligible effect on total load (Vanhatalo et al. 2013).

**Table 1:** The probability of reaching the target states set by the WFD for eastern Finnish outer archipelago (Helle et al., unpublished). *BAU* and *BSAP* business-as-usual and Baltic Sea Action Plan scenarios, respectively, *FIN3* optimistic nutrient loading reduction scenario for Finland (see Vanhatalo et al. (2013) for more information)

	Scenario		
	BAU	BSAP	FIN3
DIN	0	0	0
DIP	0.055	0.206	0.056
Chl-*a*	0	0	0
Secchi	1	1	1
Biomass	0	0	0

The probability of reaching the target states is opposite for Secchi depth and chl-*a* (Table 1). There are two main explanations for this. For practical reasons, we have used the same transformation from chl-*a* to Secchi in all segments of the GoF area, whereas the exact form and strength of the dependence between these variables likely varies among and within the WFD areas (Fernandes et al. 2012; Fleming-Lehtinen and Laamanen 2012). Another explanation may be that the WFD targets are relatively more stringent for chl-*a* than Secchi. Such nonconformity of targets is also the likely reason why the probability to meet chl-*a* targets remains zero even though the probability to meet DIP targets increases under the BSAP option. The model predicts clear decrease in chl-*a* concentration if BSAP were implemented, but these levels remain still above the target state.

### Updating Knowledge

**Fig. 3:**
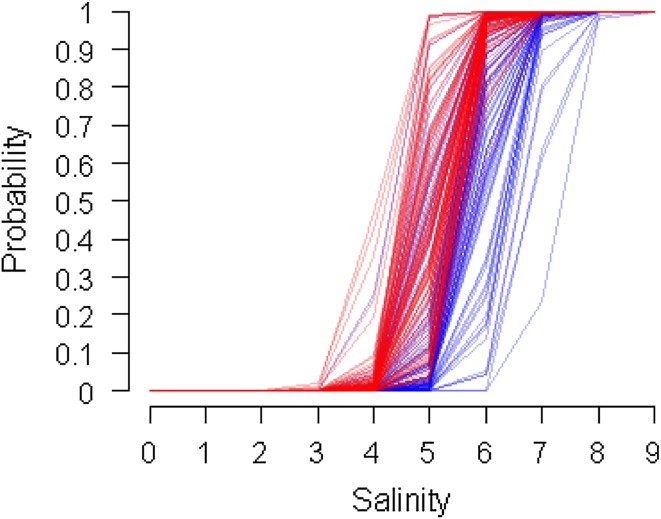
The prior and posterior understanding of the salinity threshold influencing herring growth. The logistic relationship indicates the probability for growth rate being above the modeled base level. The *blue lines* are realizations of the prior distribution; the *red lines* are realizations of the posterior distribution

Another advantage of the using Bayesian models is their ability to combine previous knowledge with new knowledge in a coherent manner, i.e., they “learn” by using prior knowledge and new data to calculate probabilistic posterior estimates (Fig. 3). In IBAM, the GoF herring stock dynamics model (Rahikainen et al., unpublished) was applied to update the available knowledge. Essentially, the model estimates the influence of eutrophication, oil spills, and harvesting on the stock dynamics, including reproduction, growth, and survival. The model output is probabilistic, and two key variables, herring catch and population biomass, were used as input in the integrative BBN (Helle et al., unpublished).

In addition to offering input to the integrative decision model, the herring model updates our understanding of several factors affecting herring population dynamics. The tanker Antonio Gramsci accident induced additional mortality of GoF herring in 1987, especially at early life-stage (Rahikainen et al., unpublished). Although one can conclude that the Antonio Gramsci oil reduced the GoF herring abundance, uncertainty about the level of mortality is high.

Further, the current status of eutrophication is estimated to substantially reduce reproduction of herring (Rahikainen et al., unpublished). In terms of chl-*a* concentration, which is used as an index for all eutrophication-related changes in the ecosystem, chl-*a* concentration should be decreased by about 50 % from the present level to facilitate the maximum reproduction of herring at any given spawning stock size.

### Challenges and Future Outlook

Bayesian models were applied in the project to provide justified uncertainty estimates and to utilize the available data complemented with the existing scientific literature to obtain an integrated risk analysis. The use of prior information in parameter estimates is important from the point of view of effective learning in science: the information content of previous publications can be used to decrease uncertainty in future analyses by including this knowledge to prior probabilities of model parameters. In particular, this is useful when examining oil accidents or other rarely occurring phenomena.

An external challenge is to introduce Bayesian approach to arenas, where the classical frequentist approaches have conventionally been applied. Among natural sciences, the ideal of objective science has dominated, and this has affected managers who may prefer “exact” advice instead of subjective probability distributions in decision-making. However, it is evident that inclusion of uncertainty is an essential part of a successful decision-making process. Point estimates oblige decision makers to be risk-neutral, which may lead to poor decisions in case the decision makers are actually risk averse (Burgman 2005). Communicating uncertainty to decision makers and stakeholders is undoubtedly a key challenge. The science community has to further develop approaches for unfolding the uncertainties in an understandable and a realistic way. Ability to consider implications of uncertainties from the policy point of view should be a specific concern. Bayesian inference describes uncertainty by probability distributions, which we regard as intuitively understandable expression of ambiguity. Thus, we strongly advocate using probabilistic approaches in risk assessment and management modeling, albeit it may be more time-demanding than the more traditional methods.

The model described in this article and other similar kinds of BBNs can assist managers in taking management decisions related to complex environmental problems. It can also be used for prioritizing future research topics through a value-of-information (VoI) analysis. This means that the posterior distributions of the model built in the IBAM project could be used to analyze whether efforts should be directed to research focusing on eutrophication, oil spills, or the fisheries management. Such a VoI analysis shows where uncertainty can be reduced with least costs (e.g., Mäntyniemi et al. 2009).

Although we find BBNs to be a flexible tool to integrate different types of knowledge and submodels, there were also some issues to be solved during the project. The major challenges were related to developing the population models, and to combine several modeling techniques coherently, e.g., the use of deterministic modeling results in a probabilistic context. Regarding the population models, a large number of prior probabilities needed to be elicited from the literature and expert knowledge, and several computational problems needed to be solved.

It is also important to notice that major uncertainties exist not only within natural systems but also on the human-side of the management, i.e., the behavior of people and their commitment to management decisions is difficult to predict (e.g., Nichols et al. 1995; Haapasaari et al. 2007; Fulton et al. 2011; Levontin et al. 2011). The relevant aspects in human behavior include stakeholder involvement, their attitudes and values, communication of knowledge and uncertainties, empowerment, and development of trust and commitment. For improved management evaluations, there is a need for models taking into account a number of aspects in human behavior.
